# Perspective: Challenges and Future Directions in Clinical Research with Nuts and Berries

**DOI:** 10.1016/j.advnut.2023.07.010

**Published:** 2023-08-02

**Authors:** Michelle L. Zuelch, Marcela D. Radtke, Roberta R. Holt, Arpita Basu, Britt Burton-Freeman, Mario G. Ferruzzi, Zhaoping Li, Neil F. Shay, Barbara Shukitt-Hale, Carl L. Keen, Francene M. Steinberg, Robert M. Hackman

**Affiliations:** 1 Department of Nutrition, University of California, Davis, CA, United States; 2 Department of Kinesiology and Nutrition Sciences, School of Integrated Health Sciences, University of Nevada, Las Vegas, NV, United States; 3 Department of Food Science and Nutrition, Illinois Institute of Technology, Chicago, IL, United States; 4 Department of Pediatrics, Arkansas Children's Nutrition Center, University of Arkansas for Medical Sciences, Little Rock, AR, United States; 5 UCLA Center for Human Nutrition, David Geffen School of Medicine at UCLA, Los Angeles, CA, United States; 6 Department of Food Science and Technology, Oregon State University, Corvallis, OR, United States; 7 Jean Mayer USDA Human Nutrition Research Center on Aging, Tufts University, Boston, MA, United States; 8 Department of Internal Medicine, University of California, Davis, CA, United States

**Keywords:** nuts, berries, walnuts, strawberries, bioactive compounds, polyphenols, phytonutrients, precision nutrition

## Abstract

Consumption of nuts and berries are considered part of a healthy eating pattern. Nuts and berries contain a complex nutrient profile consisting of essential vitamins and minerals, fiber, polyunsaturated fatty acids, and phenolics in quantities that improve physiological outcomes. The spectrum of health outcomes that may be impacted by the consumptions of nuts and berries includes cardiovascular, gut microbiome, and cognitive, among others. Recently, new insights regarding the bioactive compounds found in both nuts and berries have reinforced their role for use in precision nutrition efforts. However, challenges exist that can affect the generalizability of outcomes from clinical studies, including inconsistency in study designs, homogeneity of test populations, variability in test products and control foods, and assessing realistic portion sizes. Future research centered on precision nutrition and multi-omics technologies will yield new insights. These and other topics such as funding streams and perceived risk-of-bias were explored at an international nutrition conference focused on the role of nuts and berries in clinical nutrition. Successes, challenges, and future directions with these foods are presented here.

**Statement of significance**

The consumption of nuts and berries has been linked to improvements in a multitude of clinical outcomes related to cardiovascular function, oxidant defense, and gut health. This review summarizes recent studies, challenges, emerging areas of research, and future directions presented at the Nuts and Berries Conference on 5–6 May, 2022 at the University of California, Davis.

## Introduction

The 2020–2025 Dietary Guidelines for Americans encourages the intake of a variety of plant-based foods including nuts and berries [1]. With the goal of increasing current knowledge on nuts and berries, as well as addressing research challenges and opportunities, the *Nuts and Berries Conference: Pathways to Oxidant Defense, Vascular Function, and Gut Microbiome Changes* was held on 5 to 6 May, 2022 at the University of California, Davis. Tree nuts and berries were selected as the focus of the conference for their unique composition, bioactivity, and multitude of associated health-promoting qualities (TABLE 1, TABLE 2, TABLE 3) [[2], [3], [4], [5]]. With over 50 different edible nut species and hundreds of berry varietals, the following were selected for the purpose of the conference and this review: walnuts, almonds, hazelnuts, cashews, pecans, pistachios, strawberries, blueberries, raspberries, and blackberries.

**TABLE 1:** Flavonoids content of select nuts and berries [2**]**

Food item	Class	Flavonoid	Mean quantity (mg/100 g edible portion)
Nuts			
Walnut	Anthocyanidins	Cyanidin	2.71
Almond	Anthocyanidins	Cyanidin	2.46
	Flavan-3-ols	(-)-Epicatechin	0.6
		(-)-Epigallocatechin	2.59
		(+)-Catechin	1.28
	Flavanones	Eriodictyol	0.25
		Naringenin	0.43
	Flavonols	Isorhamnetin	2.64
		Kaempferol	0.39
		Quercetin	0.36
Hazelnut	Anthocyanidins	Cyanidin	6.71
	Flavan-3-ols	(-)-Epicatechin	0.22
		(-)-Epigallocatechin	2.78
		(-)-Epigallocatechin 3-gallate	1.06
		(+)-Catechin	1.19
Cashew	Flavan-3-ols	(-)-Epicatechin	0.93
		(-)-Epicatechin 3-gallate	0.15
		(+)-Catechin	0.90
Pecan	Anthocyanidins	Cyanidin	10.74
		Delphinidin	7.28
	Flavan-3-ols	(-)-Epicatechin	0.82
		(-)-Epigallocatechin	5.63
		(-)-Epigallocatechin 3-gallate	2.30
		(+)-Catechin	7.24
Pistachio	Anthocyanidins	Cyanidin	7.33
	Flavan-3-ols	(-)-Epicatechin	0.83
		(-)-Epigallocatechin	2.05
		(-)-Epigallocatechin 3-gallate	0.40
		(+)-Catechin	3.57
	Flavonols	Quercetin	1.46
Berries			
Strawberry	Anthocyanidins	Cyanidin	1.68
		Delphinidin	0.31
		Malvidin	0.01
		Pelargonidin	24.85
		Peonidin	0.05
		Petunidin	0.11
	Flavan-3-ols	(-)-Epicatechin	0.42
		(-)-Epicatechin 3-gallate	0.15
		(-)-Epigallocatechin	0.78
		(-)-Epigallocatechin 3-gallate	0.11
		(+)-Catechin	3.11
		(+)-Gallocatechin	0.03
	Flavanones	Naringenin	0.26
	Flavonols	Kaempferol	0.50
		Myricetin	0.04
		Quercetin	1.11
Blueberry	Anthocyanidins	Cyanidin	8.46
		Delphinidin	35.43
		Malvidin	67.59
		Peonidin	20.29
		Petunidin	31.53
	Flavan-3-ols	(-)-Epicatechin	0.62
		(-)-Epigallocatechin	0.66
		(+)-Catechin	5.29
		(+)-Gallocatechin	0.12
	Flavones	Luteolin	0.20
	Flavonols	Kaempferol	1.66
		Myricetin	1.30
		Quercetin	7.67
Raspberry	Anthocyanidins	Cyanidin	45.77
		Delphinidin	1.32
		Malvidin	0.13
		Pelargonidin	0.98
		Peonidin	0.12
		Petunidin	0.31
	Flavan-3-ols	(-)-Epicatechin	3.52
		(-)-Epigallocatechin	0.46
		(-)-Epigallocatechin 3-gallate	0.54
		(+)-Catechin	1.31
	Flavonols	Kaempferol	0.06
		Quercetin	1.05
Blackberry	Anthocyanidins	Cyanidin	99.95
		Pelargonidin	0.45
		Peonidin	0.21
	Flavan-3-ols	(-)-Epicatechin	4.66
		(-)-Epigallocatechin	0.10
		(-)-Epigallocatechin 3-gallate	0.68
		(+)-Catechin	37.06
	Flavonols	Kaempferol	0.27
		Myricetin	0.67
		Quercetin	3.58

**TABLE 2:** Phenolic acid content of select nuts and berries [3]

Food item	Class	Phenolic acid	Mean quantity (mg/100 g fresh weight)
Nuts			
Walnut	Hydroxybenzoic acids	Ellagic acid	28.5
Almond	Hydroxybenzoic acids	4-Hydroxybenzoic acid	0.00410
		Protocatechuic acid	0.26
		Vanillic acid	0.17
Hazelnut	—	—	—
Cashew	—	—	—
Pecan	—	—	—
Pistachio	—	—	—
Berries			
Strawberry	Hydroxybenzoic acids	4-Hydroxybenzoic acid 4-O-glucoside	1.53
		5-O-Galloylquinic acid	0.05
		Ellagic acid	1.24
		Ellagic acid glucoside	2.85
	Hydroxycinnamic acids	5-Caffeoylquinic acid	1.93
		Caffeoyl glucose	0.10
		Cinnamic acid	0.22
		Feruloyl glucose	0.10
		p-Coumaric acid	0.21
		p-Coumaric acid 4-O-glucoside	0.15
		p-Coumaroyl glucose	4.36
Blueberry	Hydroxybenzoic acids	4-Hydroxybenzoic acid 4-O-glucoside	0.55
		Gallic acid 4-O-glucoside	0.50
		Protocatechuic acid 4-O-glucoside	0.40
	Hydroxycinnamic acids	3-Caffeoylquinic acid	0.60
		4-Caffeoylquinic acid	0.35
		5-Caffeoylquinic acid	131.18
		5-Feruloylquinic acid	0.75
		5-p-Coumaroylquinic acid	0.35
		Caffeic acid 4-O-glucoside	0.30
		Ferulic acid 4-O-glucoside	0.55
		p-Coumaric acid 4-O-glucoside	0.95
Raspberry	Hydroxybenzoic acids	Ellagic acid	2.12
		Ellagic acid acetyl-arabinoside	0.20
		Ellagic acid acetyl-xyloside	0.36
		Ellagic acid arabinoside	2.27
		Lambertianin C	30.84
		Sanguiin H-6	76.10
	Hydroxycinnamic acids	5-Caffeoylquinic acid	0.57
		p-Coumaric acid	0.000230
		p-Coumaric acid 4-O-glucoside	0.32
Blackberry	Hydroxybenzoic acids	4-Hydroxybenzoic acid 4-O-glucoside	1.13
		Ellagic acid	43.67
		Gallic acid	4.67
		Galloyl glucose	0.27
		Protocatechuic acid 4-O-glucoside	0.43
	Hydroxycinnamic acids	3-Caffeoylquinic acid	4.53
		3-Feruloylquinic acid	0.30
		3-p-Coumaroylquinic acid	0.37
		4-Caffeoylquinic acid	0.10
		5-Caffeoylquinic acid	0.10
		Caffeoyl glucose	0.50
		Feruloyl glucose	0.43
		p-Coumaric acid 4-O-glucoside	0.27
		p-Coumaroyl glucose	0.67

**TABLE 3:** Carotenoid content of select nuts and berries [4]

Food item	Carotenoid	Mean quantity (μg/100 g edible portion)
Nuts		
Walnut	β-Carotene	12
	Lutein/zeaxanthin	9
Almond	β-Carotene	1
	Lutein/zeaxanthin	1
Hazelnut	α-Carotene	3
	β-Carotene	11
	Lutein/zeaxanthin	92
Cashew	Lutein/zeaxanthin	22
Pecan	β-Carotene	29
	β-Cryptoxanthin	9
	Lutein/zeaxanthin	17
Pistachio	α-Carotene	10
	β-Carotene	305
	Lutein/zeaxanthin	2903
**Berries**		
Strawberry	β-Carotene	7
	Lutein/zeaxanthin	26
Blueberry	β-Carotene	32
	Lutein/zeaxanthin	80
Raspberry	α-Carotene	16
	β-Carotene	12
	Lutein & zeaxanthin	136
Blackberry	β-Carotene	128
	Lutein & zeaxanthin	118

Tree nuts and berries are significant commodities in the United States. The total value of tree nuts grown in California in 2021 was estimated at $8.961 billion (almonds $5.028 billion; pistachios $2.911 billion; walnuts $1.022 billion) [5]. The total value of berries grown in California in 2021 was approximately $3.667 billion (strawberries $3.023 billion; raspberries $420,700 million; blueberries $223,500 million) [5]. With over two-thirds of US tree nuts and berries grown in California [5], the agricultural land-grant institution of the University of California, Davis was the appropriate location to convene this conference of leading researchers, registered dietitians, community partners, and industry representatives.

Regular tree nut and berry consumption is associated with a decreased risk for the development of cardiovascular disease along with favorable effects on brain and gut health [[6], [7], [8], [9], [10]]. Tree nuts provide protein and fiber and monounsaturated and polyunsaturated fatty acids, along with vitamins, minerals, and bioactive carotenoids, phytosterols, phenolics and flavonoids, and lignan and tannins, such as the condensed proanthocyanidins (particularly in pecans) and hydrolysable ellagitannins (particularly in walnuts) [11]. Berries are also a significant source of fiber and vitamin C, along with bioactive carotenoids, phenolics, including proanthocyanins and ellagitannins, and anthocyanins that provide berry color [[12], [13], [14]]. Moreover, berries provide flavan-3-ols in quantities up to 37 mg/100 g serving (TABLE 1, TABLE 2, TABLE 3), which would contribute to a recently proposed daily recommended intake level of 400 to 600 mg/d [15].

Although research results to date have been promising, mechanisms of action in general, and for vascular and gut health specifically, have yet to be fully defined. More data are needed that can be generalized to diverse population groups as well as for modeling of precision nutrition recommendations. This paper will review the progress and challenges of current nut and berry research and suggest future directions for the field.

## Research on Nuts and Berries: Successes and Challenges

### Study design

Many different study designs have been used to assess the effects of nuts and berries on cardiometabolic health. The strengths and limitations of various clinical nutrition study designs have been addressed elsewhere [16]. A summary of the past 5 y of studies on nuts and berries on outcome measures of cardiovascular and gut health is presented in TABLE 2, TABLE 3, TABLE 4, TABLE 5, TABLE 6, TABLE 7, TABLE 8, TABLE 9 [[17], [18], [19], [20], [21], [22], [23], [24], [25], [26], [27], [28], [29], [30], [31], [32], [33], [34], [35], [36], [37], [38], [39], [40], [41], [42], [43], [44], [45], [46], [47], [48], [49], [50], [51], [52], [53], [54], [55], [56], [57], [58], [59], [60], [61]] and TABLE 10, TABLE 11, TABLE 12, TABLE 13 [[62], [63], [64], [65], [66], [67], [68], [69], [70], [71], [72], [73], [74], [75]], respectively. Eligible studies consisted of clinical human trials in children, adolescents, and adults published within the last 5 y (2017–2023), exploring associations between the consumption of nuts and berries and associated biomarkers of interest.

**TABLE 4:** Intake of walnuts on cardiovascular and gut health, 2017–2023

Reference	Study design	Study duration	Subject characteristics	*n*	Nut type, quantity	Control	Relevant outcomes
**Cardiovascular health**							
Bamberger 2017 [17]	Randomized, controlled, crossover trial	8 wk	Healthy males and females (mean age 63 y)	194	Walnut, 43 g/d	Exclusion of walnuts	↓ TC^1^, non-HDL-C^2^, LDL-C∗, TG∗, ApoB^2^
Bhardwaj 2018 [18]	Randomized, controlled, crossover trial	PP HFM	OW males and females (mean age 42 y)	27	Walnut, 60 g	Almond, 77 g	↑ FMD^1^ ↓ sVCAM-1^1^
Holscher 2018 [19]	Randomized, crossover, controlled-feeding trial	3 wk	Healthy males and females (mean age 53 y)	18	Walnut, 42 g/d	Isocaloric diet, excluding walnuts	↓ *LDL-C*^1^
Alibabaie 2019 [20]	Randomized, controlled trial	4 wk	Female undergraduate students (mean age 20 y)	48	Walnut, 40 g/d	Exclusion of walnuts	↓ LDL-C^1^, TG^1^
Borkowski 2019 [21]	Randomized, controlled trial	4 wk	Hypercholesterolemic, postmenopausal females (mean age 60 y)	20	Walnut, 40 g/d	Walnut, 5 g/d	↑ lipoprotein ALA and epoxides^2^
Domènech 2019 [22]	Randomized, controlled trial	2 y	Healthy, elderly males and females (mean age 69 y)	236	Walnut, 30–60 g/d (15% energy)	Usual diet, excluding walnuts	↓ SysBP^1^
Hwang 2019 [23]	Randomized, controlled, crossover trial	16 wk	Males and females with MetS (mean age 39 y)	84	Walnut, 45 g/d	Isocaloric snack	↑ HDL-C^1^
Sanchis 2019 [24]	Randomized, crossover, controlled-feeding trial	30 d	Males and females with CKD stage 3 or 4 (mean age 71 y)	13	Walnut, 30 g/d	Isocaloric diet, excluding walnuts	↓ LDL-C^1^, SysBP^1^
Tindall 2019 [25]	Randomized, crossover, controlled-feeding trial	6 wk	OW males and females at risk of CVD (mean age 44 y)	36	Walnut, 57–99 g/d	(1) Walnut fatty acid–matched diet (2) oleic acid replaces ALA diet	↓ Central DiaBP^1^, Central and brachial MAP^1^, TC^2^, LDL-C^2^, HDL-C^2^, non-HDL-C^1^, TD:HDL-C^1^
Abdrabalnabi 2020 [26]	Randomized, controlled trial	2 y	Healthy, elderly males and females (mean age 69 y)	625	Walnut, 30–60 g/d (15% energy)	Usual diet, excluding walnuts	↓ TG^1^ HDL-C^2^
Tindall 2020 [27]	Randomized, crossover, controlled-feeding trial	6 wk	OW males and females at risk of CVD (mean age 44 y)	34	Walnut, 57–99 g/d	(1) Walnut fatty acid–matched diet (2) oleic acid replaces ALA diet	↓ TC^2^ non-HDL-C^2^, LDL-C^2^
Rajaram 2021 [28]	Randomized, controlled trial	2 y	Healthy, elderly males and females (mean age 69 y)	628	Walnut, 30–60 g/d (15% energy)	Usual diet, excluding walnuts	↓ TC^1^, LDL-C^1^, IDL-C^1^
Herselman 2022 [29]	Randomized, controlled trial	16 wk	Healthy male and female undergraduate students (mean age 22 y)	60	Walnut, 56 g/d	Usual diet, excluding walnuts	No Δ in TC or TG
**Gut health**							
Bamberger 2018 [30]	Randomized, controlled, crossover trial	8 wk	Healthy males and females (mean age 63 y)	194	Walnut, 43 g/d	Exclusion of walnuts	↑*Ruminococcaceae*,^1^*Bifidobacteria*^1^ ↓ *Clostridium*^1^
Holscher 2018 [19]	Randomized, crossover, controlled-feeding trial	3 wk	Healthy males and females (mean age 53 y)	18	Walnut, 42 g/d	Isocaloric diet, excluding walnuts	↑ *Faecalibacterium*^1^*, Clostridium*^1^*, Dialister*^1^, *Roseburia*^1^ ↓ *Ruminococcus*^1^*, Dorea*^1^*, Oscillospira*^1^, *Bifidobacterium*^1^*, SBA*^1^
García-Mantrana 2019 [31]	Nonrandomized, short-term dietary intervention trial	3 d	Healthy males and females (mean age 40 y)	27	Walnut, 33 g/d	N/A	UM-B: ↑ *Blautia*^1^*, Bifidobacterium*^1^*, Gordonibacter*^1^ UM-A: ↓ Lachnospiraceae^1^ Both*:* ↑ *Coprococcus*^1^ and *Collinsella*^1^ ↑ SCFA^1^
Tindall 2020 [32]	Randomized, crossover, controlled-feeding trial	6 wk	OW males and females at risk of CVD (mean age 44 y)	42	Walnut, 57–99 g/d	(1) Walnut fatty acid–matched diet (2) oleic acid replaces ALA diet	↑ *Roseburia*^1^*, Eubacterium eligensgroup*^1^*, Lachnospiraceae*^1^*, Gordonibacter*^1^

Includes human clinical trials that focus on only one functional food (ie, a single type of nut or berry) with outcomes of known physiologically relevant measures related to vascular function and gut health over the past 5 y (2017–2023). Excludes interventions using mixtures of different types of nuts or berries, nut- or berry-containing meals, and nut or berry extracts or oils. Also excludes interventions where nut or berry intake was in combination with other potentially confounding factors (ie, diet or lifestyle modifications including physical activity and dietary counseling).

Abbreviations: AIx, augmentation index; BP, blood pressure; CKD, chronic kidney disease; DiaBP, diastolic blood pressure; FMD, flow-mediated dilation; HDL-C, HDL cholesterol; HRV, heart rate variability; ICAM-1, intercellular adhesion molecule-1; IDL-C, IDL cholesterol; LAC, large artery compliance; LDL-C, low-density lipoprotein cholesterol; LDL:HDL, LDL cholesterol to HDL-cholesterol ratio; MAP, mean arterial pressure; MetS, metabolic syndrome; N/A, not applicable; OW, overweight; oxLDL, oxidized low-density lipoprotein cholesterol; PP HFM, postprandial high fat meal; PSV, brachial artery peak systolic velocity; SAC, small artery compliance; SBA, secondary bile acids; sVCAM-1, soluble vascular cell adhesion molecule-1; SysBP, systolic blood pressure; TC, total cholesterol; TC:HDL, total cholesterol to HDL-cholesterol ratio; T2DM, type 2 diabetes mellitus; TG, triglycerides; TMAO, trimethylamine N-oxide; UM, urolithin metabotype; VLDL-C, VLDL cholesterol.

1 denotes statistical significance <0.05.

2 denotes statistical significance <0.001.

**TABLE 5:** Intake of almonds on cardiovascular and gut health, 2017–2023

Reference	Study design	Study duration	Subject characteristics	*n*	Nut type, quantity	Control	Relevant outcomes
**Cardiovascular health**							
Lee 2017 [33]	Randomized, 4-period crossover, controlled-feeding trial	4 wk	OW and obese males and females (mean age 46 y)	31	Almond, 42.5 g/d	Isocaloric diet, excluding almonds	↓ TC^1^, non-HDL-C^1^, LDL-C^1^, ApoB^1^, SysBP^1^, DiaBP^1^ No Δ in FMD
Liu 2017 [34]	Randomized, controlled trial	16 wk	Healthy males and females (mean age 26 y)	169	Almond, 56 g/d, (1) premeal or (2) between meals	Isocaloric snack	↓ TC^1^, LDL-C^1^, non-HDL-C^1^
Bhardwaj 2018 [18]	Randomized, controlled, crossover trial	PP HFM	OW males and females (mean age 42 y)	27	Almond, 77 g	Walnut, 60 g	↓ sVCAM^1^ ↑ FMD (nonsignificant)
Dhillon 2018 [35]	Randomized, controlled trial	8 wk	Healthy males and females (mean age 18 y)	73	Almond, 56.7 g/d	Isocaloric snack	↓ TC^1^, HDL-C^1^, ^1^LDL-C No Δ in RHI, AIx, BP
Jung 2018 [36]	Randomized, controlled, crossover trial	4 wk	OW and obese males and females (mean age 52 y)	84	Almond, 56 g/d	Isocaloric snack	↓ TC^1^, LDL-C^1^, non-HDL-C^1^
Liu 2018 [37]	Randomized, controlled trial	20 wk	Healthy males and females (mean age 27 y)	85	Almond, 56 g/d	Isocaloric snack	↓ DiaBP^1^, TC^2^, HDL-C^2^, LDL-C^2^, non-HDL-C^2^, TG^2^, VLDL-C^2^
Bowen 2019 [38]	Randomized, controlled trial	8 wk	OW and obese males and females at risk for T2DM (mean age 61 y)	76	Almond, 56 g/d	Isocaloric snack	Women only: ↓ TC:HDL-C ratio^1^
Coates 2020 [39]	Randomized, controlled trial	12 wk	OW and obese males and postmenopausal females (mean age 65 y)	128	Almond, 15% energy	Isocaloric snack	↓ TG^1^, SysBP^1^ No Δ in ICAM-1, VCAM-1, SAC or LAC
Dikariyanto 2020 [40]	Randomized, controlled trial	6 wk	Males and females at risk for CVD (mean age 56 y)	105	Almond, 20% of energy	Isocaloric snack	↑ FMD^2^ ↓ LDL-C^1^, non-HDL-C^1^ No Δ TG, HDL-C or BP
Dikariyonto 2020 [41]	Randomized, controlled trial	6 wk	Males and females at risk for CVD (mean age 56 y)	105	Almond, 20% of energy	Isocaloric snack	↑ HRV^1^
Palacios 2020 [42]	Randomized, controlled, crossover trial	6 wk	OW and obese males and females with prediabetes (mean age 48 y)	33	Almond, 85 g/d	Isocaloric snack	↑ApoA^1^, HDL3-C^1^
**Gut health**							
Holscher 2018 [43]	Randomized, 5-arm crossover, controlled-feeding trial	3 wk	Healthy males and females (mean age 57 y)	18	(1) Almond, 42 g/d (whole), (2) Almond, 42 g/d (roasted), (3) Almond, 42 g/d (roasted, chopped) (4) Almond butter, 42 g/d	Exclusion of almonds	Chopped: ↑ *Lachnospira*^1^, *Roseburia*^1^, *Oscillospira*^1^ Whole: ↑ *Dialist*er^1^
Dhillon 2019 [44]	Randomized, controlled trial	8 wk	Healthy males and females (mean age 18 y)	73	Almond, 56.7 g/d	Isocaloric snack	↑ alpha-diversity^1^ ↓ *Bacteroides fragilis*^1^
Choo 2021 [45]	Randomized, controlled trial	8 wk	OW and obese males and females at risk for T2DM (mean age 61 y)	69	Almond, 56 g/d	Isocaloric snack	↑ *Ruminococcaceae*^1^
Creedon 2022 [46]	3-arm, parallel-design randomized, controlled trial	4 wk	Healthy males and females (mean age 28 y)	79	Almond, 56 g/d	Isocaloric snack	↑ SCFA (butyrate)^1^ No Δ in bifidobacteria

Includes human clinical trials that focus on only one functional food (ie, a single type of nut or berry) with outcomes of known physiologically relevant measures related to vascular function and gut health over the past 5 y (2017–2023). Excludes interventions using mixtures of different types of nuts or berries, nut- or berry-containing meals, and nut or berry extracts or oils. Also excludes interventions where nut or berry intake was in combination with other potentially confounding factors (ie, diet or lifestyle modifications including physical activity and dietary counseling).

Abbreviations: AIx, augmentation index; BP, blood pressure; CKD, chronic kidney disease; DiaBP, diastolic blood pressure; FMD, flow-mediated dilation; HDL-C, HDL cholesterol; HRV, heart rate variability; ICAM-1, intercellular adhesion molecule-1; IDL-C, IDL cholesterol; LAC, large artery compliance; LDL-C, low-density lipoprotein cholesterol; LDL:HDL, LDL cholesterol to HDL-cholesterol ratio; MAP, mean arterial pressure; MetS, metabolic syndrome; OW, overweight; oxLDL, oxidized low-density lipoprotein cholesterol; PP HFM, postprandial high fat meal; PSV, brachial artery peak systolic velocity; SAC, small artery compliance; SBA, secondary bile acids; sVCAM-1, soluble vascular cell adhesion molecule-1; SysBP, systolic blood pressure; TC, total cholesterol; TC:HDL, total cholesterol to HDL-cholesterol ratio; T2DM, type 2 diabetes mellitus; TG, triglycerides; TMAO, trimethylamine N-oxide; UM, urolithin metabotype; VLDL-C, VLDL cholesterol.

1 denotes statistical significance <0.05.

2 denotes statistical significance <0.001.

**TABLE 6:** Intake of hazelnuts on cardiovascular and gut health, 2017–2023

Reference	Study design	Study duration	Subject characteristics	*n*	Nut type, quantity	Control	Relevant outcomes
**Cardiovascular health**							
Adamo 2018 [47]	Randomized, controlled trial	2 wk	Healthy males and females (mean age 26 y)	61	(1) Peeled hazelnut paste, 30 g/d (2) Unpeeled hazelnut paste, 30 g/d	(1) Snack with peeled hazelnut paste, 30 g/d (2) Snack with cocoa powder, 2.5 g/d (3) Snack with peeled hazelnut paste, 30 g/d, and cocoa powder, 2.5 g/d	↑ HDL-C^1^, PSV^1^ ↓ LDL-C^1^, TC:HDL^2^, LDL:HDL^2^
Deon 2018 [48]	Randomized, controlled trial	8 wk	Children and adolescents with hyperlipidemia (mean age 12 y)	66	(1) Roasted, peeled, hazelnut, 15–30 g/d (2) Roasted, unpeeled hazelnut, 15–30 g/d	Dietary advice for hyperlipidemia	↓ LDL-C^2^ ↑ HDL:LDL^2^ No Δ in BP
Di Renzo 2017 [49]	Randomized, controlled, crossover trial	PP HFM	Healthy males and females (mean age 31 y)	22	Hazelnut, 40 g	HFM, no hazelnuts	↓ oxLDL^1^
Santi 2017 [50]	Randomized, controlled, crossover trial	6 wk	Healthy males and females (median age 55 y)	24	Hazelnut, 40 g	Standard/habitual diet, no hazelnuts	↓ LDL-C^1^
Tey 2017 [51]	Randomized, crossover trial	28 d	Healthy males and females (mean age 46 y)	72	Raw hazelnut, 30 g/d	Dry roasted, lightly salted hazelnut, 30 g/d	↑ HDL-C^2^, ApoA^1^, TC:HDL^2^, SysBP^1^
Guaraldi 2018 [52]	Randomized, controlled trial	8 wk	Children and adolescents with hyperlipidemia (mean age 12 y)	60	(1) Roasted, peeled, hazelnut, 15–30 g/d (2) Roasted, unpeeled hazelnut, 15–30 g/d	Dietary advice for hyperlipidemia	↓ Oxidatively-induced DNA strand breaks^1^ No Δ in oxLDL
Michels 2018 [53]	Pre-post intervention trial	16 wk	Healthy males and females (mean age 63 y)	32	Hazelnut, 57 g/d	Subject’s respective baseline data	↓ LDL-C^1^, TC:HDL^1^ No Δ in TG, HDL-C or BP
Di Renzo 2019 [54]	Pre-post intervention trial	6 wk	Healthy males and females (mean age 52 y)	24	Hazelnut, 40 g	Subject’s respective baseline data	↓ TC^1^, LDL-C^1^, TC:HDL^1^ No Δ in BP
**Gut health**							
Gargari 2018 [55]	Randomized, controlled trial	8 wk	Children and adolescents with hyperlipidemia (mean age 11 y)	15	Roasted, unpeeled hazelnut, 15–30 g/d	Normolipidemic children and adolescents	↑ Fecal acetate^1^ No Δ in α- or β-diversity

Includes human clinical trials that focus on only one functional food (ie, a single type of nut or berry) with outcomes of known physiologically relevant measures related to vascular function and gut health over the past 5 y (2017–2023). Excludes interventions using mixtures of different types of nuts or berries, nut- or berry-containing meals, and nut or berry extracts or oils. Also excludes interventions where nut or berry intake was in combination with other potentially confounding factors (ie, diet or lifestyle modifications including physical activity and dietary counseling).

Abbreviations: AIx, augmentation index; BP, blood pressure; CKD, chronic kidney disease; DiaBP, diastolic blood pressure; FMD, flow-mediated dilation; HDL-C, HDL cholesterol; HRV, heart rate variability; ICAM-1, intercellular adhesion molecule-1; IDL-C, IDL cholesterol; LAC, large artery compliance; LDL-C, low-density lipoprotein cholesterol; LDL:HDL, LDL cholesterol to HDL-cholesterol ratio; MAP, mean arterial pressure; MetS, metabolic syndrome; OW, overweight; oxLDL, oxidized low-density lipoprotein cholesterol; PP HFM, postprandial high fat meal; PSV, brachial artery peak systolic velocity; SAC, small artery compliance; SBA, secondary bile acids; sVCAM-1, soluble vascular cell adhesion molecule-1; SysBP, systolic blood pressure; TC, total cholesterol; TC:HDL, total cholesterol to HDL-cholesterol ratio; T2DM, type 2 diabetes mellitus; TG, triglycerides; TMAO, trimethylamine N-oxide; UM, urolithin metabotype; VLDL-C, VLDL cholesterol.

1 denotes statistical significance <0.05.

2 denotes statistical significance <0.001.

**TABLE 7:** Intake of cashews on cardiovascular and gut health, 2017–2023

Reference	Study design	Study duration	Subject characteristics	*n*	Nut type, quantity	Control	Relevant outcomes
Cardiovascular health							
Mah 2017 [56]	Randomized, crossover, controlled-feeding trial	4 wk	Males and females with/at risk for elevated LDL-C (mean age 56 y)	51	Cashew, 28–64 g/d (11% of energy)	Isocaloric diet, excluding cashews	↓ TC^1^, LDL-C^1^, non-HDL-C^1^, TC:HDL^1^
Baer 2019 [57]	Randomized, crossover, controlled-feeding trial	4 wk	OW males and females (mean age 57 y)	42	Cashew, 42 g/d	Isocaloric diet, excluding cashews	No significant Δ in lipid profile, BP, AIx, endothelin, adhesion molecules, or clotting factors
Damavandi 2019 [58]	Randomized, crossover, controlled-feeding trial	8 wk	Males and females with T2DM (mean age 54 y)	50	Cashew, ∼28 g/d (10% of energy)5	Isocaloric diet, excluding cashews	↓ LDL:HDL^1^

Includes human clinical trials that focus on only one functional food (ie, a single type of nut or berry) with outcomes of known physiologically relevant measures related to vascular function and gut health over the past 5 y (2017–2023). Excludes interventions using mixtures of different types of nuts or berries, nut- or berry-containing meals, and nut or berry extracts or oils. Also excludes interventions where nut or berry intake was in combination with other potentially confounding factors (ie, diet or lifestyle modifications including physical activity and dietary counseling).

Abbreviations: AIx, augmentation index; BP, blood pressure; CKD, chronic kidney disease; DiaBP, diastolic blood pressure; FMD, flow-mediated dilation; HDL-C, HDL cholesterol; HRV, heart rate variability; ICAM-1, intercellular adhesion molecule-1; IDL-C, IDL cholesterol; LAC, large artery compliance; LDL-C, low-density lipoprotein cholesterol; LDL:HDL, LDL cholesterol to HDL-cholesterol ratio; MAP, mean arterial pressure; MetS, metabolic syndrome; OW, overweight; oxLDL, oxidized low-density lipoprotein cholesterol; PP HFM, postprandial high fat meal; PSV, brachial artery peak systolic velocity; SAC, small artery compliance; SBA, secondary bile acids; sVCAM-1, soluble vascular cell adhesion molecule-1; SysBP, systolic blood pressure; TC, total cholesterol; TC:HDL, total cholesterol to HDL-cholesterol ratio; T2DM, type 2 diabetes mellitus; TG, triglycerides; TMAO, trimethylamine N-oxide; UM, urolithin metabotype; VLDL-C, VLDL cholesterol.

1 denotes statistical significance <0.05.

**TABLE 8:** Intake of pecans on cardiovascular and gut health, 2017–2023

Reference	Study design	Study duration	Subject characteristics	*n*	Nut type, quantity	Control	Relevant outcomes
Cardiovascular health							
McKay 2018 [59]	Randomized, crossover, controlled-feeding trial	4 wk	OW or obese males and females (mean age 63 y)	26	Pecan, ∼42.5 g/d (15% of energy)	Isocaloric diet, excluding pecans	↓ E-selectin^1^ ↓ TC and LDL-C trending significant No Δ in BP
Guarneiri 2021 [60]	Randomized, controlled trial	8 wk, PP HFM	Males and females at risk for CVD (mean age 48 y)	56	Pecans, 68 g/d (1) added to diet (2) substituted for isocaloric snack	Exclusion of pecans	↓ TC^1^, LDL-C^1^, TG^1^, TC:HDL^1^, non-HDL-C^1^, ApoB^1^ No Δ in BP

Includes human clinical trials that focus on only one functional food (ie, a single type of nut or berry) with outcomes of known physiologically relevant measures related to vascular function and gut health over the past 5 y (2017–2023). Excludes interventions using mixtures of different types of nuts or berries, nut- or berry-containing meals, and nut or berry extracts or oils. Also excludes interventions where nut or berry intake was in combination with other potentially confounding factors (ie, diet or lifestyle modifications including physical activity and dietary counseling).

Abbreviations: AIx, augmentation index; BP, blood pressure; CKD, chronic kidney disease; DiaBP, diastolic blood pressure; FMD, flow-mediated dilation; HDL-C, HDL cholesterol; HRV, heart rate variability; ICAM-1, intercellular adhesion molecule-1; IDL-C, IDL cholesterol; LAC, large artery compliance; LDL-C, low-density lipoprotein cholesterol; LDL:HDL, LDL cholesterol to HDL-cholesterol ratio; MAP, mean arterial pressure; MetS, metabolic syndrome; OW, overweight; oxLDL, oxidized low-density lipoprotein cholesterol; PP HFM, postprandial high fat meal; PSV, brachial artery peak systolic velocity; SAC, small artery compliance; SBA, secondary bile acids; sVCAM-1, soluble vascular cell adhesion molecule-1; SysBP, systolic blood pressure; TC, total cholesterol; TC:HDL, total cholesterol to HDL-cholesterol ratio; T2DM, type 2 diabetes mellitus; TG, triglycerides; TMAO, trimethylamine N-oxide; UM, urolithin metabotype; VLDL-C, VLDL cholesterol.

1 denotes statistical significance <0.05.

**TABLE 9:** Intake of pistachios on cardiovascular and gut health, 2017–2023

Reference	Study design	Study duration	Subject characteristics	*n*	Nut type, quantity	Control	Relevant outcomes
Gut health							
Hernández-Alonso 2017 [61]	Randomized, controlled, crossover trial	4 mo	Males and females with prediabetes (mean age 55 y)	39	Pistachio, 57 g/d	Isocaloric diet, excluding pistachios	↓ Gut microbiota-related metabolites (hippurate, p-cresol sulfate, dimethylamine)^1^ and TMAO^1^

Includes human clinical trials that focus on only one functional food (ie, a single type of nut or berry) with outcomes of known physiologically relevant measures related to vascular function and gut health over the past 5 y (2017–2023). Excludes interventions using mixtures of different types of nuts or berries, nut- or berry-containing meals, and nut or berry extracts or oils. Also excludes interventions where nut or berry intake was in combination with other potentially confounding factors (ie, diet or lifestyle modifications including physical activity and dietary counseling).

Abbreviations: AIx, augmentation index; BP, blood pressure; CKD, chronic kidney disease; DiaBP, diastolic blood pressure; FMD, flow-mediated dilation; HDL-C, HDL cholesterol; HRV, heart rate variability; ICAM-1, intercellular adhesion molecule-1; IDL-C, IDL cholesterol; LAC, large artery compliance; LDL-C, low-density lipoprotein cholesterol; LDL:HDL, LDL cholesterol to HDL-cholesterol ratio; MAP, mean arterial pressure; MetS, metabolic syndrome; OW, overweight; oxLDL, oxidized low-density lipoprotein cholesterol; PP HFM, postprandial high fat meal; PSV, brachial artery peak systolic velocity; SAC, small artery compliance; SBA, secondary bile acids; sVCAM-1, soluble vascular cell adhesion molecule-1; SysBP, systolic blood pressure; TC, total cholesterol; TC:HDL, total cholesterol to HDL-cholesterol ratio; T2DM, type 2 diabetes mellitus; TG, triglycerides; TMAO, trimethylamine N-oxide; UM, urolithin metabotype; VLDL-C, VLDL cholesterol.

1 denotes statistical significance <0.05.

**TABLE 10:** Intake of strawberries on cardiovascular and gut health, 2017–2023

Reference	Study design	Study duration	Subject characteristics	*n*	Berry type, quantity	Control	Relevant outcomes
**Cardiovascular health**							
Feresin 2017 [62]	Randomized, double-blind, controlled, parallel-arm trial	8 wk	Postmenopausal females (mean age 59 y) with pre- or stage 1 hypertension	60	FDSP, 25 g/d or 50 g/d	Control powder	25 g/d: ↓ SysBP^1^ and PWV^1^ 50 g/d: ↑ NO metabolites^1^
Holt 2020 [63]	Randomized, controlled, double-blind, crossover trial	Acute (1 h), Short-term (1 wk)	Adolescent males (mean age 16 y)	25	FDSP, 50 g/d	Isocaloric control powder, devoid of polyphenols	↑ plasma nitrate and nitrite^2^ and RHI^1^ No Δ in platelet reactivity
Basu 2021 [64]	Randomized, controlled crossover trial	14 wk	Males and females (mean age 53 y) with one or more characteristics of MetS	33	FDSP, 13 g/d or 32 g/d	Isocaloric control powder	↓ Total VLDL and chylomicrons^2^, small VLDL^2^, and total and small LDL particles^2^ No Δ in conventional lipid profile
Huang 2021 [65]	Randomized, controlled, double-blinded, 2-arm, 2-period crossover trial	4 wk	Males and females (mean age 53 y) with moderate hypercholesterolemia	34	FDSP, 25 g/d	Isocaloric control powder	↑ FMD (treatment-by-hour effect)^1^ ↓ SysBP^1^ No Δ in lipid profile, ApoA, or ApoB
**Gut health**							
Ezzat-Zadeh 2021 [66]	Single arm intervention trial (placebo group omitted due to prebiotic content)	4 wk	Males and females (mean age 30 y)	14	FDSP, 26 g/d	N/A	↑ *20 OTUs*^1^ ↓ *4 OTUs*^1^ No Δ in fecal microbial metabolites or SCFA

Includes human clinical trials that focus on only one functional food (ie, a single type of nut or berry) with outcomes of known physiologically relevant measures related to vascular function and gut health over the past 5 y (2017–2023). Excludes interventions using mixtures of different types of nuts or berries, nut- or berry-containing meals, and nut or berry extracts or oils. Also excludes interventions where nut or berry intake was in combination with other potentially confounding factors (ie, diet or lifestyle modifications including physical activity and dietary counseling).

Abbreviations: AIx, augmentation index; ALA, alpha-linolenic acid; BP, blood pressure; FDBP, freeze-dried blueberry powder; FDRP, freeze-dried raspberry powder; FDSP, freeze-dried strawberry powder; FMD, flow-mediated dilation; HDL-C, high-density lipoprotein cholesterol; LDL-C, low-density lipoprotein cholesterol; LDL:HDL, LDL cholesterol to HDL-cholesterol ratio; MetS, metabolic syndrome; N/a, not applicable; OTU, operational taxonomic unit; OW, overweight; PWV, pulse wave velocity; SCFA, short-chain fatty acids; SysBP, systolic blood pressure; TC, total cholesterol; TC:HDL, total cholesterol to HDL-cholesterol ratio; T2DM, type 2 diabetes mellitus.

1 denotes statistical significance <0.05.

2 denotes statistical significance <0.001.

**TABLE 11:** Intake of blueberries on cardiovascular and gut health, 2017–2023

Citation	Study design	Study duration	Subject characteristics	*n*	Berry type, quantity	Control	Relevant outcomes
Cardiovascular health							
Curtis 2019 [67]	Double-blind, randomized controlled trial	6 mo	OW and obese males and females (mean age 63 y) with MetS	115	Blueberry, 75 g/d or 150 g/d	Control powder	↑FMD^1^, AIx^1^, HDL-C^1^, ApoA^1^ No Δ PWV, BP, LDL-C or LDL:HDL ratio
Stote 2020 [68]	Double-blind, randomized, controlled trial	8 wk	Males with T2DM (mean age 67 y)	52	Blueberry, 22 g/d FDBP	Control powder	No Δ in TC, LDL-C, HDL-C, or BP
Curtis 2022 [69]	Double blind, randomized controlled trial	Acute (1 dose)	OW and obese males and females (mean age 63 y) with MetS	45	FDBP, 26 g/d (1 C whole fruit equivalent)	Isocaloric control powder	↑HDL-C^1^, ApoA^1^ ↓ TC^1^ No Δ in LDL-C, TG, FMD, PWV, AIx, or BP
Krikorian 2022 [70]	Randomized, controlled trial	12 wk	OW and obese males and females (mean age 56 y)	27	FDBP, ½ C whole fruit equivalent	Control powder devoid of fiber	No Δ in lipid profile
Wang 2022 [71]	Randomized, controlled. crossover trial	1 wk	Normal to OW males and females (mean age 26 y)	37	Blueberry, 160 g/d FDBP, 20 g/d	Fiber matched control capsule	No Δ in lipid profile, plasma nitrite, PWV, or BP

Includes human clinical trials that focus on only one functional food (ie, a single type of nut or berry) with outcomes of known physiologically relevant measures related to vascular function and gut health over the past 5 y (2017–2023). Excludes interventions using mixtures of different types of nuts or berries, nut- or berry-containing meals, and nut or berry extracts or oils. Also excludes interventions where nut or berry intake was in combination with other potentially confounding factors (ie, diet or lifestyle modifications including physical activity and dietary counseling).

Abbreviations: AIx, augmentation index; ALA, alpha-linolenic acid; BP, blood pressure; FDBP, freeze-dried blueberry powder; FDRP, freeze-dried raspberry powder; FDSP, freeze-dried strawberry powder; FMD, flow-mediated dilation; HDL-C, high-density lipoprotein cholesterol; LDL-C, low-density lipoprotein cholesterol; LDL:HDL, LDL cholesterol to HDL-cholesterol ratio; MetS, metabolic syndrome; OTU, operational taxonomic unit; OW, overweight; PWV, pulse wave velocity; SCFA, short-chain fatty acids; SysBP, systolic blood pressure; TC, total cholesterol; TC:HDL, total cholesterol to HDL-cholesterol ratio; T2DM, type 2 diabetes mellitus.

1 denotes statistical significance <0.05.

**TABLE 12:** Intake of blackberries on cardiovascular and gut health, 2017–2023

Citation	Study design	Study duration	Subject characteristics	*n*	Berry type, quantity	Control	Relevant outcomes
Cardiovascular health							
Solverson 2018 [72]	Randomized, controlled, crossover trial	1 wk	OW or obese males (mean age 60 y)	27	Blackberry, 600 g/d	Isocaloric gelatin	↑ fat oxidation^1^ No Δ in TG

Includes human clinical trials that focus on only one functional food (ie, a single type of nut or berry) with outcomes of known physiologically relevant measures related to vascular function and gut health over the past 5 y (2017–2023). Excludes interventions using mixtures of different types of nuts or berries, nut- or berry-containing meals, and nut or berry extracts or oils. Also excludes interventions where nut or berry intake was in combination with other potentially confounding factors (ie, diet or lifestyle modifications including physical activity and dietary counseling).

Abbreviations: AIx, augmentation index; ALA, alpha-linolenic acid; BP, blood pressure; FDBP, freeze-dried blueberry powder; FDRP, freeze-dried raspberry powder; FDSP, freeze-dried strawberry powder; FMD, flow-mediated dilation; HDL-C, high-density lipoprotein cholesterol; LDL-C, low-density lipoprotein cholesterol; LDL:HDL, LDL cholesterol to HDL-cholesterol ratio; MetS, metabolic syndrome; OTU, operational taxonomic unit; OW, overweight; PWV, pulse wave velocity; SCFA, short-chain fatty acids; SysBP, systolic blood pressure; TC, total cholesterol; TC:HDL, total cholesterol to HDL-cholesterol ratio; T2DM, type 2 diabetes mellitus.

1 denotes statistical significance <0.05.

**TABLE 13:** Intake of raspberries on cardiovascular and gut health, 2017–2023

Citation	Study design	Study duration	Subject characteristics	*n*	Berry type, quantity	Control	Relevant outcomes
Cardiovascular health							
Istas 2018 [73]	3-arm double-blind, randomized, controlled, crossover trial	Acute (24 h)	Males (mean age 27 y)	10	Raspberry, 200 g/d or 400 g/d	Control drink devoid of polyphenols	↑ FMD^1^ No Δ PWV, AIx, or BP
Xiao 2019 [74]	Randomized, single-blind, three-arm, 24-h, within-subject crossover trial	Acute, postprandial	OW or obese males and females (mean age 34 y) with prediabetes and insulin resistance	32	Raspberry, 125 g/d or 250 g/d	Absence of raspberry with test meal	No Δ in TG
Franck 2020 [75]	2-arm parallel-group, randomized, controlled trial	8 wk	Males and pre-menopausal females (mean age 32 y)	48	Raspberry, 280 g/d	Habitual diet	↓ SysBP^2^, ApoB^2^ No Δ in lipid profile
Zhang 2022 [74]	Randomized crossover trial	4 wk	OW or obese males and females (mean age 35 y) with prediabetes and insulin resistance	36	Raspberry, 50 g/d	N/A	↓ TC^2^, LDL-C^2^, and LDL:HDL ratio^2^
Gut Health Outcomes							
Zhang 2022 [74]	Randomized crossover trial	4 wk	OW or obese males and females (mean age 35 y) with prediabetes and insulin resistance	36	Raspberry, 50 g/d	N/A	↑ *Eubacterium eligens*^2^↓ Ruminococcus gnavus^2^

Includes human clinical trials that focus on only one functional food (ie, a single type of nut or berry) with outcomes of known physiologically relevant measures related to vascular function and gut health over the past 5 y (2017–2023). Excludes interventions using mixtures of different types of nuts or berries, nut- or berry-containing meals, and nut or berry extracts or oils. Also excludes interventions where nut or berry intake was in combination with other potentially confounding factors (ie, diet or lifestyle modifications including physical activity and dietary counseling).

Abbreviations: AIx, augmentation index; ALA, alpha-linolenic acid; BP, blood pressure; FDBP, freeze-dried blueberry powder; FDRP, freeze-dried raspberry powder; FDSP, freeze-dried strawberry powder; FMD, flow-mediated dilation; HDL-C, high-density lipoprotein cholesterol; LDL-C, low-density lipoprotein cholesterol; LDL:HDL, LDL cholesterol to HDL-cholesterol ratio; MetS, metabolic syndrome; N/A, not applicable; OTU, operational taxonomic unit; OW, overweight; PWV, pulse wave velocity; SCFA, short-chain fatty acids; SysBP, systolic blood pressure; TC, total cholesterol; TC:HDL, total cholesterol to HDL-cholesterol ratio; T2DM, type 2 diabetes mellitus.

1 denotes statistical significance <0.001.

2 denotes statistical significance <0.05.

Two long-term intervention trials, the PREDIMED (Prevención con Dieta Mediterránea) and the COcoa Supplement and Multivitamin Outcomes Study (COSMOS), published in 2018 and 2022, respectively, provide examples of study designs that could be useful for future planning. The PREDIMED dietary intervention trial provides the strongest evidence to date that incorporation of nuts into a healthy Mediterranean dietary pattern in individuals ages 55 to 80 y old for 4.8 y can reduce risk of cardiovascular events (myocardial infarction, stroke, and cardiovascular death) by 28% [9]. The COSMOS trial demonstrated that the daily intake of monomeric and polymeric flavanols from cocoa in older adults (men >60 y and women >65 y of age) reduces risk for cardiovascular morbidity and mortality [76]. Although the COSMOS study utilized a flavanol supplement compared to a whole food, it is a case study to support the need for larger trials with clinical outcomes based on the use of multi-site data of surrogate outcomes from dietary interventions that use randomized, double-blind controlled trials in crossover or parallel-arm study designs for studies of nuts or berries [[77], [78], [79]].

A common study design for whole foods is the replacement of the test food with a nutritionally matched, isocaloric substitute. However, matching nutritional content can be a challenge because food processing, such as blending berries and roasting nuts, causes a disruption to the nutrient matrix, potentially changing the bioavailability of key nutrients [[80], [81], [82]]. For nuts, controls often include the complete omission of the nut of interest. For berry research, a number of considerations exist that are alternative to consuming the whole food. One is the use of freeze-dried berry powders as the test product, controlled with an isocaloric powder either lower or devoid of potential bioactives. Attempts have been made to mask the control powders, but issues such as product color, texture, scent, and mouth feel are challenging to completely match. Although this approach is similar to a classical pharmaceutical trial design, blinding study personnel and participants is challenging, thus creating both performance and detection bias. Additionally, freeze-dried berry powders can have a different food matrix compared to the whole food, which could influence outcome measures as well as limit generalizability to the whole fruit. A second approach for berry research is the encapsulation of test and control powders. This can aid in participant masking, but the total amount of test product provided can be limiting, and large intakes of control gelatin capsules have resulted in adverse effects [81,82]. A third option can be examining 2 or more intake levels, with or without a true control group [21,83]. Finally, the use of macro- and micronutrient matched gummies with similar amounts of calories, sugars, and fiber, but devoid of other bioactives, is a novel option for use as a comparative control. In all of these approaches, the potential bioactivity of the control itself must be considered. For example, isocaloric control powders that are low in polyphenols may still have a considerable amount of fiber in order to obtain similar mouth feel and texture, but the fiber content may have effects on lipid metabolism and the microbiome, which could influence outcome measures.

Multiple cultivars of berries exist, some of which have differences in the content of bioactive ingredients, thus limiting comparison and extrapolation of results [83,84]. For nuts, walnuts contain a variety of phenolic acids, catechins, and flavonoids, most of which have been reported to possess bioactivity. Significant differences in the concentration of 16 phenolic compounds were identified when comparing black (*Juglans nigra L.*) and English (*Juglans regia L.*) walnuts [21,84]. More than 50 cultivars of strawberries exist in the United States. To help reduce the potential experimental variability created with the use of different cultivars, the California Strawberry Commission has produced a freeze-dried test material that utilizes a composite of genotypes to produce a powder that is characterized for its macro- and micronutrients and bioactive components [63]. The US Highbush Blueberry Council also provides a powder that is a 50/50 mixture of 2 cultivars (*Vaccinium corymbosum* and *Vaccinium virgatum*) [85]. A limitation of this approach is that the standardized mixture may contain varieties with reduced or low bioactivity. However, the advantage of this approach is that the composite represents the “market basket” available to consumers and allows comparison of results from studies conducted among different research groups and generalizability of results to a broader berry application actually used by consumers.

In addition to cultivar differences, factors such as climate and seasonal differences due to heat, sunlight, and rainfall can contribute additional variability. Given the above, the characterization of bioactives within these foods is critical. New analytical equipment and techniques have increased the precision of food composition compared to analyses performed decades ago. Current advances in the development of nutrition databases have been reviewed elsewhere [86]. For example, databases such as that from the USDA FoodCentral could be strengthened if the date of the analyses was included, along with the protocols used and the number of samples analyzed. Linking resources from repositories detailing data, such as chemical composition and bioactivity, will help both plant scientists and health professionals to make accurate and timely recommendations and guide future research.

### Individual variability

Free-living populations have differences in background diets that can influence their responses to the intake of test foods, potentially creating significant variation in baseline measurements. This variability presents a challenge when elucidating clinically relevant effects, especially if unknown a priori, where statistical significance can be masked by combining and analyzing groups together. Interindividual variability may be mitigated by increasing sample size as well as using a crossover design, but challenges in recruitment, retention, and budget constraints exist. One way to help minimize experimental variability is through a run-in period to identify participants who may be differentially metabolizing bioactive phenolics or with the goal of minimizing or removing potentially confounding metabolites from circulation prior to the intervention [13]. However, study designs that employ highly controlled settings, strict inclusion and exclusion criteria, extended washout periods that alter background diets, and ask participants to follow an atypical consumption pattern does not reflect “normal” life and may have limited applicability to the general population. Another useful model that also has limitations is the provision of nuts or berries in amounts and duration that are greater than normally consumed. Feeding relatively high amounts of nuts or berries for a limited period of time has been employed to demonstrate proof-of-concept and provide a basis for further exploration for changes in physiology [63], cognitive performance [87], and gut microbiome profiles [88]. Subsequent study designs must be realistic, guided by the USDA FoodCentral database for portion size. These trial designs should also use a duration that is realistically achievable by consumers, whose food purchasing behavior can be influenced by cost, access, and seasonal availability of the food. Studies using average daily portion sizes typically require intervention periods of months, which present challenges regarding participant compliance and retention and cost of the study. In a review of 231 reports on berries and health, approximately 70% of studies used interventions of less than 3 mo or contained less than 50 participants [89]. Meeting the challenge of conducting long-term studies using amounts of foods in a typical diet, with a representative sample of participants, requires a significant commitment of resources.

The health and functional levels of participants are other factors that influence study designs and outcomes. For example, studies on cognitive performance with both nuts and berries have assessed effects among those both with and without cognitive impairments [39,90,91]. In such studies, short-term interventions may show little or no response after the addition of nuts or berries to the diet [39]. Although the net change may not be statistically significant, this model does not address the ability of the food to prevent decline, which would require long-term testing. Further, an individual with cognitive impairments might demonstrate favorable responses compared to baseline measures following nut or berry intake but may still not reach the level of performance of a healthy individual. In both instances, neither change from baseline, nor absolute values of performance, fully captures the beneficial cognitive response [[92], [93], [94]].

Dietary interventions require the incorporation of foods into an individual’s eating pattern, which may present a number of challenges. One is the creation of boredom with eating the same food on a regular basis. Second is that the caloric load of the test nut or berry may displace the intake of other nutrient-dense foods. These factors may make compliance for the entire study duration an issue, particularly if the intervention is weeks or months in duration [95]. A third challenge involves compliance. In berry research studies, compliance is often not reported, or the reported range of intake is so variable that it is hard to discern the significance of the results [96]. The use of food intake metabolite markers is an emerging tool that can help verify compliance [97].

In addition to compliance, dietary patterns are an important consideration needed for the interpretation of results because individuals do not eat a single food in the absence of other foods. Background or habitual intake is often not addressed in nutritional trials. The potential variability in habitual dietary intake of participants is often a confounding factor in nutrition research [98]. Dietary assessment methods, with 24-h recalls, 3-d food records, and food frequency questionnaires, all have limitations [99]. These subjective measures may also not accurately capture the potential for nutrient-nutrient interactions that may alter polyphenolic or other bioactive components attributed to nut and berry consumption. Further complicating this issue is the observation that study designs utilizing longer-term interventions or that require the intake of a large amount of the test food are more likely to result in overreporting food intake due to fear that participants may be dismissed from the intervention [100]. Innovations in dietary assessment methodology using “smart” eyeglasses or other image-based technologies have been proposed to address this issue [101]. Assessing the relationship between the intake of nutrients and bioactives from a whole food product to physiologic responses is difficult, as a multitude of processes are affected, including regulation of vascular function, provision of oxidant defense, and changes in gut microbiome profiles and subsequent output of secondary metabolites [10, 102]. Additionally, bioactives from nuts and berries can interact with each other as well as other dietary components to alter bioavailability and health-promoting properties [80]. For example, intake of dietary fats in conjunction with berries has been demonstrated to increase carotenoid bioavailability [80].

Results could also be confounded by dietary changes made by participants in addition to incorporation of the test nut or berry. Habitual dietary intake is often measured through food frequency questionnaires or repeated 24-h dietary recalls. However, these subjective measures may not accurately capture the potential for nutrient-nutrient interactions that may alter polyphenolic or other bioactive components attributed to nut and berry consumption. Further complicating this issue is the observation that study designs utilizing longer-term interventions or that require the intake of a large amount of the test food are more likely to result in overreporting food intake due to fear that participants may be dismissed from the intervention [103].

Expanding the scope of populations to be studied is another key area for future research. Most clinical trials using nuts and berries have been conducted in middle-aged or older Caucasian adults with one or more cardiometabolic risk factors [11,83,84,100]. Whether these results extend to other population groups is either inferred or unknown. Future research would benefit from extending the study populations to include those from other racial and ethnic groups [12]. This is particularly important in order to address the current NIH research initiative in precision nutrition and health, the “Nutrition for Precision Health powered by the All of Us Research Program” [104]. The inclusion of biological females in clinical nutrition trials is imperative, yet the current literature includes predominantly male participants [105]. Because many studies on nuts and berries focus on cardiometabolic outcomes, the unique aspects of female physiology must be considered [106]. For example, vascular function fluctuates with the phase of the menstrual cycle, which has largely been ignored in most past studies [107]. More studies are also needed in young children as well as in young adults up to about the age of 40 [108]. A pilot study (*n*=17) reported a correlation between blueberry supplementation and acute positive effects on memory and executive function (defined as significant functional improvement with statistical probability of <5% chance) in 7- to 10-y old children [109]. A large study among pregnant women-infant dyads (*n*=2208) reported positive protective neuropsychological effects on long-term cognitive development in children at 1, 5, and 8 y of age when nuts were consumed during gestation [110]. Finally, translation of research results is challenging when considering socioeconomic status (SES), particularly when food items are not accessible or affordable [111,112]. Barriers to participation in clinical research studies among those of low SES include a low interest in clinical trials, inefficient or inadequate explanation of the study in culturally appropriate terms, participants’ distrust of biomedical research, and participant burden, including lack of transportation or the inability to prioritize participation in research over work obligations [113].

### Study duration

Like many other dietary studies, research on nuts and berry studies often use acute (several hour) studies evaluating postprandial effects. However, either a lack of or successful demonstration of benefits does not necessarily predict a similar outcome over extended periods of intake. Depending on the outcome measure, detectable effects may take weeks or months for the intervention. Only a limited number of studies exist assessing the impact of nut or berry intake on the incidence or severity of diseases or metabolic dysfunction, which require durations of months or years [114].

### Moving Forward: Precision Nutrition, Multi-omics, and Biomonitoring

Precision nutrition evaluates an individual’s unique biological characteristics such as genotype and phenotype, including DNA expression, influences of the gut microbiome, and metabolic response to specific foods or dietary patterns, as well as dietary habits and external factors influencing outcomes such as social determinants of health, to determine the most effective dietary strategies to improve health and prevent disease [[115], [116], [117]]. Understanding the sources of interindividual variability that contribute to metabolic heterogeneity and applying mathematical modeling and computational algorithms will be essential to refining dietary recommendations. Several recent publications comprehensively review research gaps and study design considerations in the field of precision nutrition and specifically concerning (poly)phenolic-rich plant foods [118,119]. Precision nutrition will lead to important discoveries pertaining to interindividual responsiveness to the intake of nuts and berries. Ultimately, this information can be applied via targeted recommendations to individuals and groups for achievable and sustainable dietary intake of nuts and berries to promote optimal health.

The incorporation of biomonitoring technologies into study designs may also be used for precision nutrition. Current and emerging mobile devices can provide continuous data collection in free-living populations with minimal participant burden. The study of nuts and berries would be enhanced with the use of devices that can capture real-time physiological outputs at home that reflect normal living conditions [120]. Further collaborative efforts in the fields of bioengineering and artificial intelligence hold promise for advancing the understanding of benefits from nuts or berries.

An emerging personal biomonitoring technology is the Precision Health Toilet, which collects and evaluates human urine and stool, which are then analyzed using artificial intelligence to determine flow rate and volume of urine, as well as fecal analysis via the Bristol Stool Scale [121]. A second type of toilet seat, the Heart Seat, has recently been approved by the US Food and Drug Administration for home use to monitor heart rate and oxygen saturation, with future plans to add sensors that monitor systolic and diastolic blood pressure [122]. Assessment of metabolites in the excreta seems like a feasible goal for future development, which may be useful, for example in the detection of urinary and fecal metabolites that can reflect the metabolism of ellagic acid (from strawberries and walnuts) to urolithins [123] and of (-)-epicatechin (from a variety of berries and tree nuts) to γ-valerolactone [124]. A third example is an ingestible capsule containing a biological photosensor that can detect gut inflammation [125,126]. Bioluminescence can be monitored from bacteria that have been engineered to illuminate when they come into contact with a molecule for which they have been coded, such as urolithins from berries or lipid-sensitive metabolites from nuts. Finally, another type of ingestible capsule has recently been detailed that collects samples from multiple regions of the human intestinal tract during normal digestion. This device has been used to explore the role of the gut microbiome in physiology and disease, with novel findings that intestinal and stool metabolomes differ dramatically [127,128]. The ability of nut or berry intake to alter such metabolomes, and their association with changes in physiological function and health outcomes, would be an interesting area for future research. Although these technologies are still in their infancy, they have promise to further precision nutrition research efforts on nuts and berries.

Research addressing the issue of “responders” compared with “nonresponders” is important in understanding the metabolic discrepancies in many studies on nuts and berries. For example, platelet aggregation phenotypes can vary significantly by individual responsiveness to oxylipins, bioactive lipid mediators derived from polyunsaturated fatty acids present in nuts as well as in extra virgin olive oil [129]. Variations in circulating metabolites and microvascular function following the intake of freeze-dried strawberry powder have been reported [130]. Those individuals producing increased nitrate and nitrite levels showed favorable changes in function whereas those showing no change in nitrate or nitrite levels did not [63,130]. Another example is illustrated by a recent letter [131] in response to a systematic review [132] of almond intake and inflammatory biomarkers. The letter notes that while the review included amounts of almonds ranging from 10 to 113 g/d, favorable responses only occurred at intake of <60 g/d. Further, the authors note that although the review reports beneficial effects of almond intake on reduction in C-reactive protein and interleukin-6, subgroup analyses showed that the effects on these 2 outcomes were not significant among those with obesity or who were rated as unhealthy prior to the intervention.

Characterizing participants according to precision nutrition, including the use of genetic phenotyping to identify target genes that may result in “responders” and “nonresponders” prior to enrollment may be helpful for clinical trials but does not reflect responses in a free-living population. Furthermore, in addition to physiological variations, sociobehavioral differences among individuals that may modulate responses to berries and nuts must also considered. Nonetheless, innovative precision nutrition models that can identify interindividual differences would be useful in defining mechanisms of action and potentially who would benefit the most from regular nut or berry consumption.

Plasma and serum concentrations are useful to identify the bioavailability and bioefficacy of key nutrients and phytochemicals found in nuts and berries [133]. Some compounds, such as small molecular weight polyphenols, are first absorbed in their native state in the small intestine. Other polyphenols can be biotransformed via the host microbiota to a second set of compounds that are subsequently absorbed and confer additional bioactivity beyond that obtained from the parent molecules [134,135]. Monitoring both host and microbial metabolites in the blood and urine, and those that may accumulate in tissues of interest such as the liver and gastrointestinal epithelium, among other tissues, would be useful in understanding the dynamics of nut and berry bioactivity and specific association with site of actions [134].

Broader application of orthogonal approaches that combine untargeted with targeted metabolomic platforms and combined with the use of advanced informatics will support new understanding about the absorption, distribution, metabolism, and excretion of compounds found in nuts and berries. For example, the UC Davis West Coast Metabolomics Center conducts both targeted and untargeted assays that assess plasma microbial metabolites using a biogenic amine panel that identifies and quantifies acylcarnitines, trimethylamine N-oxide, cholines, betaines, nucleotides and nucleosides, methylated and acetylated amines, di- and oligo-peptides, and a number of microbially modified food-derived metabolites.

Some interindividual differences in response to nut or berry intake have been attributed to the composition of the gut microbiome [136]. For example, ellagitannins are polyphenolic compounds present in strawberries, raspberries, and walnuts that are metabolized by gut bacteria into an array of urolithins [88, 137]. The production of urolithins relies on the capacity of specific microbes, *Gordonibacter pamelaeae* and *Gordonibacter urolithinfaciens* [[138], [139], [140]]. Urolithins may decrease symptoms of chronic metabolic diseases, including inflammation and dyslipidemia [137]. Following a single intake of red raspberries, individuals with prediabetes and insulin resistance had lower concentrations of circulating urolithins compared to levels found in those who were metabolically healthy, a result related to gut microbiome composition [141]. In the same population, consuming red raspberries for 4 wk improved hepatic insulin resistance and total and LDL cholesterol in the prediabetes group, and the effects were related to decreased *R. gnavus* and increased *E. eligins*. Overall, including a practical amount of red raspberry in the diet regularly is a low-calorie dietary strategy that improves gut microbiota composition and function in individuals with prediabetes and insulin resistance resulting in improvements in metabolic health [74]. With a sustained emphasis on the role of gut microbiota in nutrition research, advances in our understanding of food-gut dynamics will provide new insights about the role of nuts and berries in human health and performance.

Although research on a specific nut or berry provides insight into bioactivity and potential mechanisms of action, such focus also creates the potential for fragmentation because the search for overall dietary patterns is not addressed. The composition of fruits and nuts differ at the molecular level, and a broader view assessing similarities in chemistry and health benefits is critical for translational research as well as for messaging purposes. For example, blueberries, strawberries, pomegranate, walnuts, and grapes all have reported benefits for cardiovascular health, driven largely by the presence of similar polyphenols, which are present at varying quantities in each of these foods [65,[142], [143], [144]]. Although health professionals and consumers often hear messaging on a single berry or nut, the potential benefits of increasing consumption of the broader category may be obscured or lost. This challenges the ability to maintain consistent messaging and align better with translatable dietary guidance. Future interventions that combine nuts and berries with one or more other foods within a food matrix at dietary achievable doses and in more diverse populations are warranted [[145], [146], [147]].

To date, multi-omics technologies have provided valuable insights into exposure-disease relationships [148,149]. Coupled with artificial intelligence, predictive modeling and continuous, personalized monitoring, these data-intensive outcomes can provide further insights about the health benefits associated with regular intake of nuts or berries. Use of highly personalized data collection devices will require secure data repositories [150]. One of the challenges of similar foods being studied in differing formats and by various research groups is the utility of the data as a combined set. Differences in test materials and experimental designs make integration of data difficult. The proper curation of combined data, whether physiologic, metabolomic, or genomic, is critical to ensure that combined datasets provide synergy, statistical power, and enhanced usefulness.

### Novel Markers of Health Outcomes

The cardiometabolic benefits from regular consumption of nuts or berries are widely reported and include improved vascular function [63,84,[151], [152], [153]], reduction of cardiovascular disease risk factors [154,155], improved insulin sensitivity [156,157], and reduced risk of type 2 diabetes mellitus [[158], [159], [160]]. Antioxidant [124,161] and anti-inflammatory [162,163] capacity and activity have also been noted. Metabolic outcomes may be context-specific and related to the physiologic state of the individual and host microbiome composition, among other factors. Examples include findings of ellagitannin and ellagic acid rich foods (raspberries and walnuts) resulting in differential responses in healthy individuals compared to those with prediabetes, who are dependent on gut microbial-derived metabolite profiles (urolithin metabotype) [74,88,141,164]. Many factors contribute to interindividual variability in response to diet that can extend to context-specific aspects influencing the magnitude of health benefits and reinforces the importance for further research aimed at advancing discoveries in precision nutrition. Additional health outcomes related to nut or berry intake are outlined below.

### Body composition

Adding nuts or berries to the daily diet may be advantageous for weight management for several physiological reasons. One is that these foods produce feelings of satiety, helping to reduce the desire to consume calorie-rich snacks that are low in vitamins, minerals, and fibers, ultimately improving body composition over time [165]. A second possibility is due to urolithins, secondary metabolites produced from ellagitannins in nuts and berries [137]. Urolithins increase the activation of the adenosine monophosphate-activated protein kinase (AMPK) pathway, resulting in anti-obesogenic properties *in vitro* and in animal models [166, 167]. AMPK increases fatty acid oxidation and decreases triglyceride accumulation [166]. Phosphorylation of AMPK may also decrease cholesterol synthesis and lipogenesis by downregulating 3-hydroxy-3-methylglutaryl coenzyme A reductase activity and sterol regulatory-element binding protein expression [167,168]. In clinical studies exploring the relationship between food and body composition, the incorporation of nuts and berries into the diet was associated with weight loss or maintenance [[169], [170], [171]].

### Brain health

Regular consumption of nuts or berries has been reported to support brain health and cognitive function, motor control, mood, and executive function at physiologically relevant intakes [172]. Middle-aged and older adults experienced improvements in balance, gait, and memory, and children experienced higher executive function and positive affect after acute and regular intake of both strawberries and blueberries [32,109,[173], [174], [175], [176]]. These beneficial effects may be the result of direct effects on brain signaling or indirect effects through oxidant defense and anti-inflammatory properties of polyphenols and other bioactive compounds in nuts and berry foods [[177], [178], [179]].

The gut-brain axis is an emerging area of research. Most studies are preclinical in nature using animal models but are suggestive of a significant role of gut microbial-derived ellagitannin metabolites on brain health and neuroprotection [180,181].

### Skin health

The influence of nuts and berries on skin health and appearance is an emerging area of research [182]. Regular intake of almonds, a good source of fatty acids and polyphenols, has been associated with a significant decrease in facial hyperpigmentation and wrinkle severity [183,184]. A walnut protein hydrolysate administered to rats exposed to ultraviolet radiation significantly reduced skin photoaging and enhanced skin elasticity [185]. Supplementation with ellagic acid, a compound found in many berries, prevented ultraviolet B (UVB)-related inflammation and collagen degradation related to skin wrinkling and aging in a murine model [186]. More human studies, using objective measures of skin wrinkles, skin elasticity and response to low-dose UVB radiation exposure are warranted. Monitoring skin responses to a UVB radiation challenge has been used as a marker of whole-body antioxidant status in response to almond consumption [187]. The response to a UVB challenge has also been used to monitor oxidant defenses and changes in skin microbiome following the intake of pomegranate juice [188].

### Eye health

Age-related macular degeneration (AMD) is the third leading cause of vision loss worldwide [189]. Anthocyanins, carotenoids, flavonoids, and vitamins C and E, found in many berries, have been shown to reduce risk of eye-related diseases [190,191]. Goji berries, containing the highest amount of zeaxanthin of any known food, hold particular promise since this compound binds to receptors in the macula to offer protection from blue and ultraviolet light [192]. Regular supplementation with 28 g/d of goji berries for 3 mo increased macular pigment optical density, a biomarker for AMD, as well as the skin carotenoid index [192]. Nuts may also be protective against AMD since they are a rich source of vitamin E and essential fatty acids. Regular intake of nuts has been associated with a reduced risk and slower progression of AMD in 2 epidemiological studies, thought to be due to the beneficial role of polyunsaturated fatty acids [193, 194].

## Agricultural and Administrative Challenges

### New cultivars

Identification of new cultivars with traits desirable for growers, processors, and consumers is a continuous effort. As researchers continue to produce new varieties by both conventional and molecular-driven approaches, assessing these varieties for nutritional value is a challenge. A combination of broad targeted and untargeted metabolomic approaches, along with defined functional phenotyping (ie, assays relevant to bioavailability, metabolism, or health functionality) could be used for rapid screening and defining of mechanistic pathways associated with health. However, consumer preferences for new cultivars are often driven by size and appearance of the berry or nut and flavor, rather than its nutritional value [195]. This would further confirm the need to balance improvements to nutritional profiles with enhancement of consumer-driven traits, maintaining the marketable nature of the berries and nuts.

### Funding and research bias

Biomedical research, particularly for clinical studies, is expensive and resource intensive. Although the USDA competitive grants program offers funding for outstanding research projects, budget limitations favor animal or in vitro study proposals. Compelling pilot data is needed to be competitive for clinical studies funded by the USDA or NIH, so many researchers submit their initial ideas to commodity groups representing specific nuts or berries. Commodity groups represent farmers, processors, and distributors and have been instrumental in supporting fundamental and applied research focused on their specific berry or nut.

The perception that studies funded by nut and berry commodity groups are inherently biased in favor of the test food is an issue sometimes raised by critics, journalists, and the general public. As in all nutrition research, ethical considerations regarding the structure of research questions, hypotheses, study design, outcome measures, interpretation of data, and conclusions must be rigorously considered. The food and beverage industries have played a key role in providing funds and supporting nutrition research on individual foods and beverages, including berries and nuts. Although this draws scrutiny regarding scientific integrity and data reporting, collaboration between academia and industry compared to exclusive corporate funding may help offset some of these concerns. For example, in multiple reported studies, matching funds were also provided by nonindustry sources, including institutional and federal agencies. In other cases, while the food industry provided the test agents, key research personnel and staff were not supported by the same funding source. The academia-industry collaboration has also led to the formation of scientific advisory committees that evaluate and recommend proposals for funding, a peer review process that helps ensure rigorous study designs, data reporting, and dissemination of results. Human studies of sufficient statistical power are expensive, labor-intensive efforts requiring sophisticated and costly laboratory equipment and supplies. In order for research proposals to be competitive for funding from the USDA or NIH, pilot data is required, and for nuts and berries, the only realistic source of funding for these exploratory trials is from industry sources. Critics of industry support for nutrition research have yet to propose realistic alternatives for funding needed to generate initial data. Further, ongoing industry funding of nuts and berries research has yielded important insights into the molecular and physiological understanding of mechanisms of action. Without industry support, provided in an ethical and transparent manner, advances in our understanding of the role of nuts and berries in a healthy dietary pattern would be limited.

A risk-of-bias (ROB) study of 5675 journal articles used in systematic reviews published between 1930 and 2015, representing a wide variety of nutrition topics, concluded that ROB domains started to significantly decrease after 1990, and particularly after 2000 [196]. Another study examined the incidence of favorable outcomes reported in studies funded by the food industry in the 10 most-cited nutrition and dietetics journals in 2018 [197]. Of the 1461 articles included in the analysis, 196 (13%) reported industry support, with processed food and dietary supplement manufacturers supporting 68% of the studies included. Studies supported by any nut or berry commodity group were not considered due to an incidence lower than 3% of qualifying articles. Studies with food industry support reported favorable results in 56% of their articles, compared to 10% of articles with no industry involvement. The authors offer a number of suggestions to help minimize real or perceived bias, calling on research institutions to enforce strict, regularly updated, and transparent oversight of all research projects involving industry. Suggestions in support of research transparency and integrity have also been advanced from guidelines adapted from the International Life Sciences Institute North America [198]. This served as the basis for the development of consensus guiding principles for public-private partnerships developed by a group of representatives from academia, scientific societies and organizations, industry scientists, and the USDA, NIH, US Centers for Disease Control, and the US Food and Drug Administration [199]. These provisions include full disclosure of funding and confirmation of no direct industry involvement in the study design, data and statistical analyses, and interpretation of the results and only minimal, if any, involvement of industry coauthor(s), often given as a courtesy to acknowledge funding and logistical support by the investigators with no intellectual involvement by the study sponsor [200]. This is in contrast to industry-initiated research, where the industry office or commodity group sets predetermined research objectives, provides intellectual collaboration, and often has input on the study design, interpretation of results, and decisions regarding publication [201, 202].

Although some critics may argue that repeated industry funding in support of research groups that report favorable results on a particular nut or berry shows a bias toward positive outcomes, other interpretations are also possible. First, few labs have the infrastructure, detailed methodology and analytical equipment, and trained personnel to conduct clinical studies in an efficient and timely manner. Second, registering the study on the clinicaltrials.gov research registry also provides transparency about study design, outcome measures, and results. Industry-funded studies conducted at major universities have layers of review and accountability within their organizations to guard against malfeasance, and while these layers may not focus directly on precise elements of research design and interpretation of results, faculty members at such institutions generally have a level of integrity and accountability, knowing that administrative review exists. Calls for industry-funded research are often broad in scope, which allows researchers to generate proposals, research questions, and hypotheses that do not have preconceived outcomes. A third consideration is that the nuts or berries under study may simply have sufficient bioactivity to produce favorable outcomes, independent of potential researcher bias.

## Conclusion

Nuts and berries are an important part of a healthy eating pattern. With unique nutritional profiles, including an array of bioactive compounds and phytonutrients, nuts and berries support a variety of health-promoting qualities and are associated with improved cardiometabolic, cognitive, gut microbiome, and other outcomes. Improved understanding and new insights about nuts and berries in the human diet are predicted with advances in precision nutrition and multi-omics technologies. Nonetheless, fundamental research issues exist, including study duration, testing amounts that reflect typical use and in heterogenous populations, appropriate control groups, and funding streams. The simple question: “are nuts and berries healthy?” is best answered by “it depends” on factors discussed in this review such as in whom, how much, and how often.

### Author Contributions

The authors’ responsibilities were as follows—RMH, MLZ, MDR: responsible for design; RMH, MLZ, MDR, RRH, AB, BBF, MGF, ZL, NFS, BSH, CLK, FMS: writing; RMH, MLZ, MDR, RRH, AB, BBF, MGF, ZL, NFS, BSH, CLK, FMS: responsible for final content; and all authors: read and approved the final manuscript.

### Disclaimer

The findings and conclusions in this publication have not been formally disseminated by the U. S. Department of Agriculture and should not be construed to represent any agency determination or policy.

### Conflict of Interest

The authors report no conflicts of interest.

### Funding

This research was supported in part by the intramural research program of the United States Department of Agriculture, National Institute of Food and Agriculture, AFRI A1343 Food and Human Health program, Accession No. 1026872. Additional support from unrestricted gifts was provided by the California Walnut Commission and the California Strawberry Commission.
